# Analyzing the association between functional connectivity of the brain and intellectual performance

**DOI:** 10.3389/fnhum.2015.00061

**Published:** 2015-02-10

**Authors:** Gustavo S. P. Pamplona, Gérson S. Santos Neto, Sara R. E. Rosset, Baxter P. Rogers, Carlos E. G. Salmon

**Affiliations:** ^1^InBrain Lab, Department of Physics, Faculty of Philosophy, Sciences and Letters of Ribeirão Preto, University of São PauloSão Paulo, Brazil; ^2^Faculty of Medicine of Ribeirão Preto, University of São PauloSão Paulo, Brazil; ^3^Department of Radiology and Radiological Sciences, Department of Biomedical Engineering, Institute of Imaging Science, Vanderbilt UniversityNashville, TN, USA

**Keywords:** functional connectivity, fMRI, network parameters, intelligence, Wechsler intelligence scales, exploratory data analysis

## Abstract

Measurements of functional connectivity support the hypothesis that the brain is composed of distinct networks with anatomically separated nodes but common functionality. A few studies have suggested that intellectual performance may be associated with greater functional connectivity in the fronto-parietal network and enhanced global efficiency. In this fMRI study, we performed an exploratory analysis of the relationship between the brain's functional connectivity and intelligence scores derived from the Portuguese language version of the Wechsler Adult Intelligence Scale (WAIS-III) in a sample of 29 people, born and raised in Brazil. We examined functional connectivity between 82 regions, including graph theoretic properties of the overall network. Some previous findings were extended to the Portuguese-speaking population, specifically the presence of small-world organization of the brain and relationships of intelligence with connectivity of frontal, pre-central, parietal, occipital, fusiform and supramarginal gyrus, and caudate nucleus. Verbal comprehension was associated with global network efficiency, a new finding.

## Introduction

Functional connectivity is expressed as correlations between the blood oxygenation level dependent signals in different regions of the brain (Friston et al., 1993; Biswal et al., 1995; Van den Heuvel and Hulshoff Pol, 2010). Consistent spatial patterns of functional connectivity are found for individuals at rest and are presumed to reflect information processing networks (Lowe et al., 1998; Raichle et al., 2001; Beckmann et al., 2005; Damoiseaux et al., 2006). Recent advances in neuroimaging have provided new tools to measure and analyze interactions between brain regions, catalyzing the study of functional connectivity of the brain (Van den Heuvel and Hulshoff Pol, 2010). An important recent expansion of functional connectivity studies was the use of the principles of graph theory (Watts and Strogatz, 1998) to depict the brain as an efficient complex network, with brain regions as the nodes and functional connectivity as the edge weights (Sporns and Zwi, 2004; Bullmore and Sporns, 2009). The functional brain network shows a highly efficient small-world organization, with a high level of local clustering and short effective lengths between brain regions. This leads to high global efficiency of information flow in the network (Sporns and Zwi, 2004; Van den Heuvel et al., 2008).

An important tool to measure the intelligence in adults is the Wechsler Adult Intelligence Scale (WAIS), based on the “global capacity of the individual to act purposefully, to think rationally and to deal effectively with his environment” (Wechsler, 1939). Some studies have applied intelligence indices to anatomical and functional brain measurements (Gray et al., 2003; Haier et al., 2004; Song et al., 2008; Gläscher et al., 2009; Li et al., 2009). A previous study found that higher IQ scores are associated with greater functional connectivity within a fronto-parietal network, suggesting that the coordination of these regions is an important neural basis of individual intelligence (Song et al., 2008). A region-specific analysis of the lateral prefrontal cortex, part of the fronto-parietal network, found that its global connectivity predicted working memory performance and fluid intelligence (Cole et al., 2012). Two studies have reported an association between efficiency of global communication and intellectual performance, suggesting that individuals with higher intelligence have a more organized brain network overall (Van den Heuvel et al., 2008; Song et al., 2009).

However, the relationships between brain functional connectivity and psychological measures such as intelligence are not fully defined. In the present exploratory study, we pursued this line of research further by considering how the several indices of intelligence measured by the Wechsler Adult Intelligence Scale (WAIS-III) related to connection strengths and network properties in a brain network defined by a set of 82 a priori cortical and subcortical regions derived from an atlas (Tzourio-Mazoyer et al., 2002). The use of a smaller set of regions of interest preserves structural and physiological similarities, while simplifying the analysis and easing the interpretation of the findings relative to the commonly used voxel-wise approach. In contrast to some studies that considered a priori regions known to be related to intelligence (Song et al., 2008; Cole et al., 2012), the present study explored the brain as a whole, with no region-specific or network-specific hypotheses. This analysis could help to elucidate how the human brain supports particular intellectual processes, extending previous work and providing background to future studies.

## Materials and methods

### Participants

Thirty one healthy people were recruited from the academic community and the local population living in the state of São Paulo, Brazil. They were right-handed, had no history of neurological or psychological illnesses, and were native speakers of Brazilian Portuguese. People with a range of educational levels were recruited to provide a greater range of intelligence scores (Table 1). Thirty of these participants made up Dataset 1. Volunteers participated in this study after responding to the standard screening interview of the Hospital of Clinics in Ribeirão Preto, and providing written consent as approved by the Research Ethics Committee of University of São Paulo.

**Table 1:** **Demographic data and estimated intelligence scores**.

**Category**	**Data**
Gender (M/F)	15/14
Age (years-old)	26.8 ± 5.8
Verbal IQ	111.7 ± 10.8
Performance IQ	116.0 ± 11.4
Full-scale IQ	114.2 ± 10.0
Verbal comprehension index	111.9 ± 11.0
Perceptual organization index	115.3 ± 11.9
Working memory index	111.4 ± 12.3
Processing speed index	116.1 ± 12.0

*Age and intelligence scores are shown as mean ± standard deviation*.

### Measures of individual intelligence

The level of intellectual performance was measured (Gérson S. Santos Neto and Sara R. E. Rosset) using the WAIS III test (Wechsler Adult Intelligence Scale) as modified for the Portuguese-speaking population of Brazil (Nascimento, 1998). WAIS-III is a widely used instrument that assesses several cognitive domains contributing to intelligence. It has high test-retest reliability and a large database for comparison and standardization (Gläscher et al., 2009). Measurements originating from the third version of the test are the four fundamental indices Verbal Comprehension Index, Perceptual Organization Index, Working Memory Index, and Processing Speed Index; and the overall score, Full-Scale IQ. The test took 1 h 30 min on average and was given at a separate time from the image acquisition (less than 2 months apart, except for one participant with a 3-month difference).

### Data acquisition

Resting-state functional magnetic resonance images (eyes open, no fixation) from each participant were acquired in a Phillips 3 Tesla scanner with a Quasar Dual gradient system (80 mT/m, 200 mT/m/ms), using an eight channel head coil and SENSE encoding. An EPI sequence was performed with the following parameters: 2000 ms repetition time, 30 ms echo time, 240 × 240 mm field of view, 3 × 3 mm in-plane voxel size, 4.0 mm slice thickness, 0.5 mm slice gap, 32 slices, 80° flip angle, 200 volumes, 25.2 Hz bandwidth per pixel. Overall functional acquisition time was 6:48, including four initial volumes that were discarded prior to analysis.

High-resolution anatomical images were also acquired using a 3D T1 weighted turbo-field-echo gradient sequence with the following parameters: 2500 ms repetition time, 3.2 ms echo time, 7.0 ms time echo spacing, 900 ms inversion time, 1 mm isotropic voxel size, 8° flip angle, 240 × 240 × 160 mm^3^ field of view, and overall time 5:19. Diffusion and other functional images were also acquired, but not used in the present analysis.

A separate set of resting-state functional magnetic resonance images (open eyes, with fixation) from 30 subjects (13M/17F, age: 26.5 ± 5.5, age range: 20–42, right-handed) was included in the analysis to provide a baseline for the small-worldness measurement, and classified as Dataset 2. These images were from the 1000 Functional Connectomes Project (Biswal et al., 2010), specifically the data acquired in Leipzig, Germany, in a 3 Tesla scanner with the following parameters: 2300 ms repetition time, 34 slices, 195 volumes.

### Pre-processing

Functional MRI data were processed using the SPM8 software (http://www.fil.ion.ucl.ac.uk/spm/software/spm8) and the CONN functional connectivity toolbox (14), both implemented in MatLab (R2013a, The MathWorks, Natick, MA, USA). For each individual's functional images, rigid body movement was measured and corrected using a two-step procedure in which the first of the specified functional images was used as a reference to which all subsequent images were realigned, then the functional images were re-registered to the mean image. Participants who moved more than 2 mm in translation or 1 degree in rotation were excluded from analysis. Functional images were then spatially smoothed using a Gaussian filter of 5 mm full width at half maximum.

Anatomical images from each volunteer were registered to the mean functional image created in the previous step. The anatomical volumes were segmented into gray matter, white matter and cerebrospinal fluid compartments and non-linearly registered to the MNI standard space. The resulting masks were eroded once at an isotropic voxel size of 2 mm to minimize partial volume effects. This step produced spatial normalization parameters that were used to apply the transformations to the functional images.

Voxel time series were additionally processed to reduce noise. Signals from the white matter and CSF compartments (5 principal components each) and the estimated head motion time series and first differences were removed by regression. A temporal band-pass filter was applied to remove signals outside the range 0.008–0.09 Hz (Whitfield-Gabrieli and Nieto-Castanon, 2012).

Average signals were extracted from a set of 116 regions defined by the Automated Anatomical Labeling (AAL) atlas, which is a macroanatomical parcellation of the single subject MNI-space template brain (Tzourio-Mazoyer et al., 2002). Eight of the AAL regions were excluded from the analysis due to their small size (less than 300 voxels), which increased the likelihood that partial volume effects would contaminate signals from those regions. Cerebellum and cerebellar vermis regions were also excluded because they were not fully covered by the fMRI. Therefore, 82 cortical and subcortical regions were included in total, all of them shown in the Supplemental Material (Table S1) with their AAL abbreviations and the locations of their centers, in x, y, and z.

### Analysis of functional connectivity and intelligence

**Figure 1:**
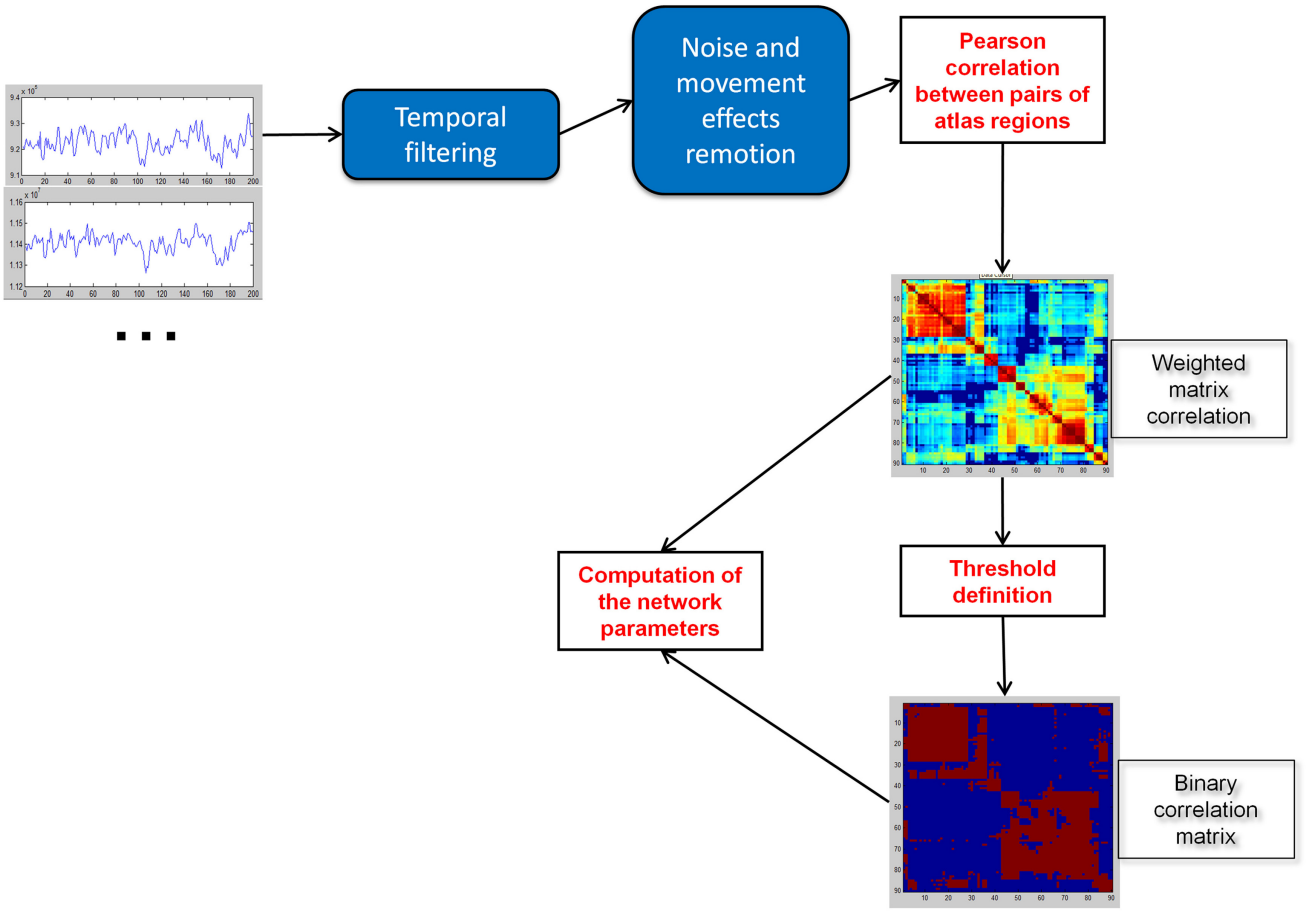
**Construction of weighted and binary correlation matrices of the brain**.

**Figure 2:**
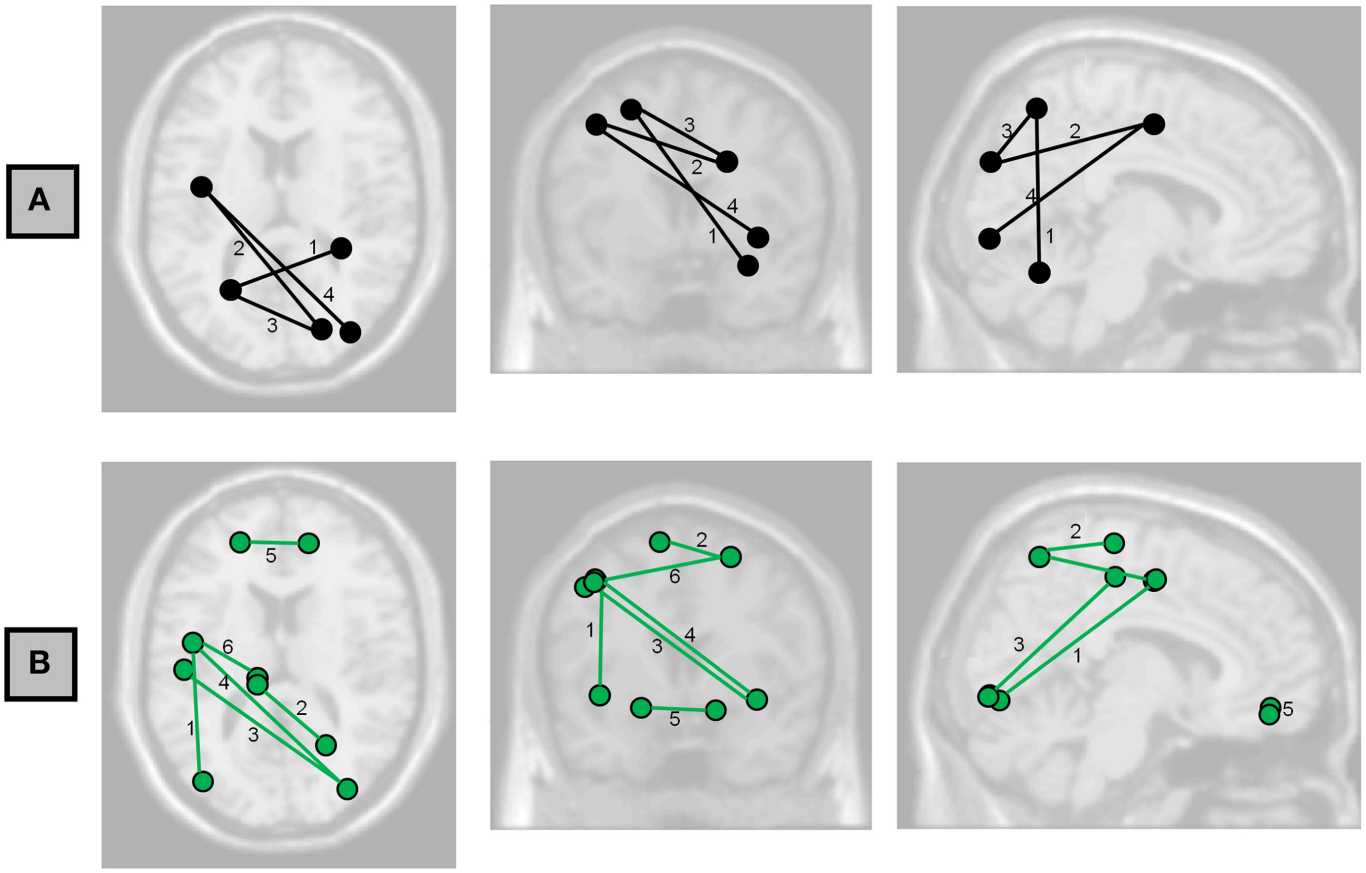
**Axial, coronal, and sagittal projections of the brain showing the functional connections having associations with (A) Full-Scale IQ and (B) Perceptual Organization Index at FDR < 0.05**. Numbers correspond to the labels in **Table 3**.

Weighted association matrices were created (Figure 1) using the Pearson correlations between the time series of each pair of brain regions. Functional connectivity of each path was compared with the four fundamental intelligence indices and the Full-Scale IQ using the Pearson correlation coefficient (**Table 3**, Figure 2). Negative values of the matrices were included to consider also the functional anticorrelations. Functional connectivity values were the Fisher Z scores computed between the time series of each pair of regions. Each list of 3321 *p*-values (all pairs of 82 regions) was adjusted to maintain a false discovery rate of 0.05, separately for each IQ index.

### Graph analysis

We examined small-worldness, characteristic path length, clustering coefficient, and global and local efficiency. Characteristic path length is the shortest path length between all pairs of nodes. Clustering coefficient is the number of connections in the neighborhood of a certain node divided by the maximum number of possible connections between the neighbors of this node. Global efficiency is inversely related to the characteristic path length and measures how efficiently information is communicated between nodes. Local efficiency of a given node is the inverse of the average shortest path connecting all neighbors of that node and evaluates the influence of different paths based on the connection weights of the node's neighbors, i.e., a path made of strong connections contributes to the local efficiency more than a path made of weak connections. Therefore, local efficiency of a node is related to its clustering coefficient, since more connections or stronger ones between neighbors directly affect both measures.

All the network parameters were computed using the Brain Connectivity Toolbox (BCT) (Rubinov and Sporns, 2010). Negative correlations in association matrices were not included in any analysis of network measures, since they need to be removed prior to BCT computations (Rubinov and Sporns, 2010, 2011). Different network measures require different pre-processing of the association matrix.

#### Small-worldness analysis

**Figure 3:**
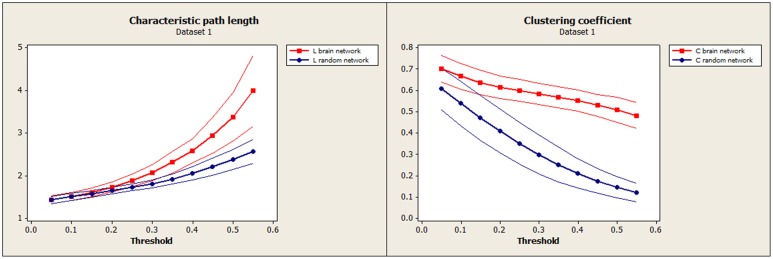
**Our data (Dataset 1): Mean characteristic path length for brain (red) and random (blue) networks are shown on the left as a function of threshold**. Mean clustering coefficient for brain (red) and random (blue) networks are shown on the right. Confidence bands represent ±1 standard deviation.

**Figure 4:**
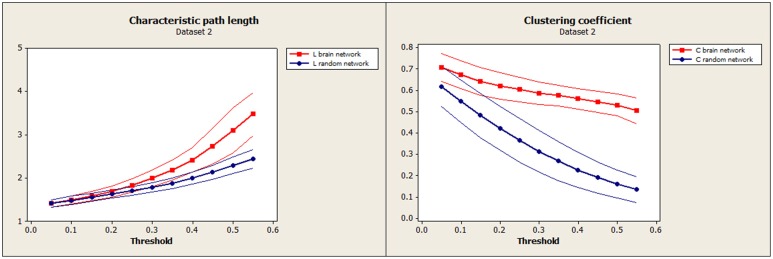
**Independent test data (Dataset 2): Mean characteristic path length for brain (red) and random (blue) networks are shown on the left**. Mean clustering coefficient for brain (red) and random (blue) networks are shown on the right. Confidence bands represent ±1 standard deviation.

Characteristic path length (L) and clustering coefficient (C) were computed to study the small-worldness of our data (Dataset 1, Figure 3) and of an independent set of resting-state fMRI (Dataset 2, Figure 4) to verify the small-worldness of the network in our sample and to provide a baseline for our measurements. These calculations used binary matrices obtained by thresholding the correlation matrices (Figure 1) at a range of values. The same analysis was applied to 20 random matrices with the same number of connections and similar distribution of connections (Sporns and Zwi, 2004), to obtain a random-matrix characteristic path length (L_random_) and clustering coefficient (C_random_). The networks are said to have small-world organization for correlation thresholds in which L = L_random_ and C > C_random_; this was calculated using a 2-sample *t*-test for *p* ≤ 0.01.

#### Analysis of global network properties and intelligence

Global network parameters (characteristic path length, clustering coefficient, and efficiency), obtained using weighted networks, were related to the intelligence indices using the Pearson correlation coefficient (**Table 4**). The Z-transformed correlation matrix was used for the association matrix, except for global efficiency, which used the Pearson correlations due to the need to restrict the range to [0,1]. Negative values were set to zero. Some form of normalization is necessary to obtain measures that are independent of the network size, dividing parameters obtained from brain networks by those obtained from random networks. For normalization of weighted networks, a recently approach purposes to compute the average value from an ensemble of surrogate graphs (Stam et al., 2009). In our study, 100 surrogate random weighted networks were constructed, derived from the original networks by randomly permuting the edge weights. The parameters of these random weighted networks were averaged and used in normalization. For this analysis, *p*-values were not adjusted.

**Figure 5:**
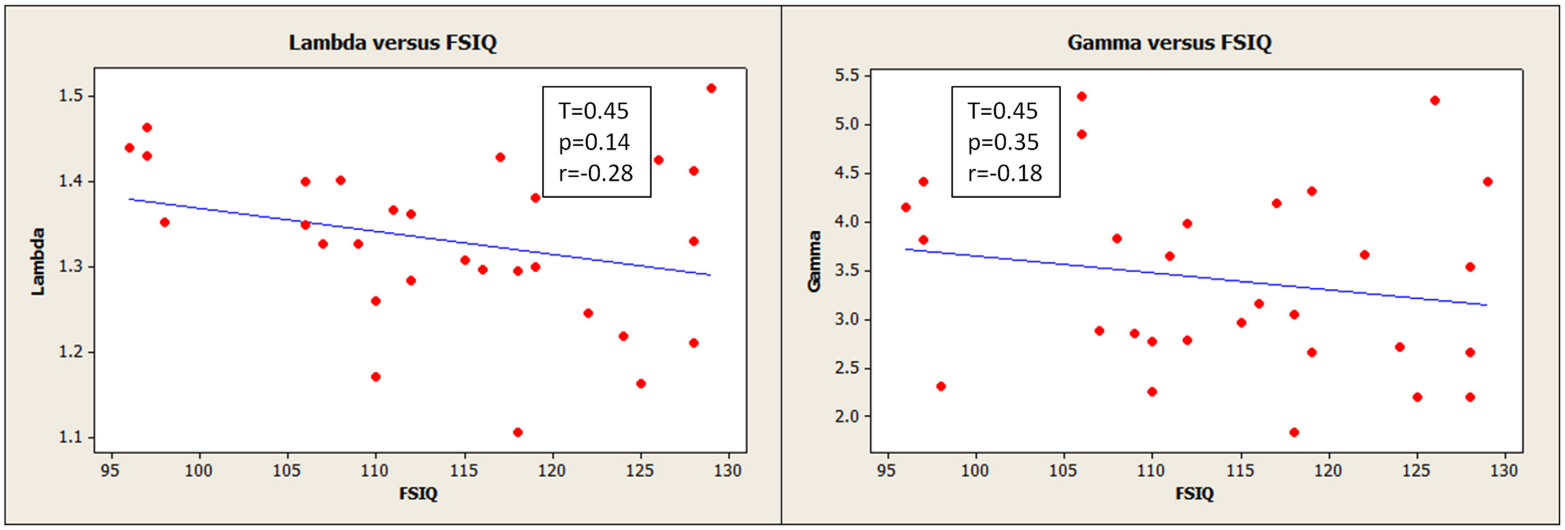
**Normalized characteristic path length (lambda) (left) and normalized clustering coefficient (gamma) (right) had slight negative relationships with Full Scale IQ, though these were not statistically robust**. The network path strengths were based on binarized correlation matrices thresholded at 0.45 for this analysis. (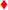) corresponds to measurements for an individual participant.

An additional analysis of global characteristic path length and global clustering coefficient associated to intelligence indices was performed using a binarized association matrix (thresholded at *r* = 0.45) to facilitate comparisons with Van den Heuvel et al. (2009) (Figure 5). Both metrics were normalized using the same 20 equivalent random binary matrices, specified in Section Small-Worldness Analysis, averaged for each brain network. Pearson correlations were also transformed using the Fisher Z in this analysis.

#### Analysis of local network properties and intelligence

**Figure 6:**
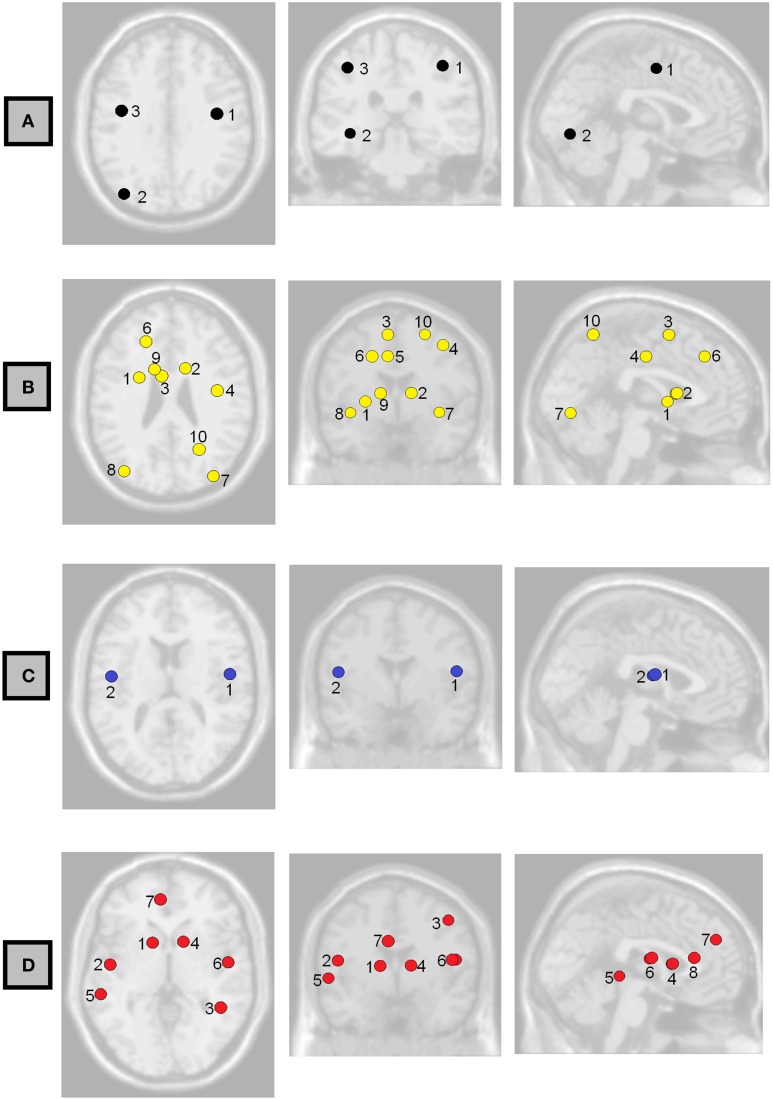
**Axial, coronal, and sagittal views of the brain showing the non-normalized weighted-network local efficiency in the regions where it had the strongest association with (A) Full-Scale IQ, (B) Verbal Comprehension Index, (C) Working Memory Index, and (D) Processing Speed Index**. Labels correspond to those shown in **Table 5**.

Finally, local efficiency, which is related to clustering coefficient, was related to the intelligence indices using the Pearson correlation coefficient (**Table 5**, Figure 6). Local efficiency calculations used the untransformed Pearson correlation matrix for the association matrix, except that negative weights were replaced with 0. For this analysis, false discovery rates were computed per node (over the list of the 81 other regions).

## Results

Of the 31 volunteers, one did not perform the intelligence test and exhibited excessive movement during imaging acquisition; thus 30 participants (Dataset 1) were included in the small-world organization study (ages: mean 27 years, standard deviation 6, range: 19–38; 15 women) and 29 participants were included in the intellectual performance study (ages: mean 27 years, standard deviation 6, range: 19–38; 14 women). Demographic data for the intellectual performance study (29 participants) are in Table 1.

We have included a table of correlations between the intelligence indices in our sample (Table 2). Verbal IQ (VIQ) was strongly correlated with Verbal Comprehension Index (VCI) and Working Memory Index (WMI). Performance IQ (PIQ) was correlated strongly with Perceptual Organization Index (POI) and moderately with Processing Speed Index (PSI). This was expected because VIQ and PIQ are derived from the fundamental indices, and so these indices were not used in the analysis of this study. Full scale IQ (FSIQ) was strongly correlated with Perceptual Organization and Working Memory indices and moderately correlated with Verbal Comprehension and Processing Speed Indices, also expected.

**Table 2:** **Correlations between intelligence scores**.

	**VIQ**	**PIQ**	**FSIQ**	**VCI**	**POI**	**WMI**
PIQ	**0. 54**					
FSIQ	**0. 90**	**0. 85**				
VCI	**0. 84**	0. 29	**0. 67**			
POI	**0. 52**	**0. 95**	**0. 81**	0. 23		
WMI	**0. 73**	**0. 56**	**0. 74**	**0. 49**	**0. 52**	
PSI	0. 45	**0. 55**	**0. 55**	0. 38	0. 35	**0. 53**

*Bold numbers represent significant values for α < 0.01*.

### Associations between functional connectivity and intelligence

Possible correlations of functional connectivity with FSIQ and perceptual organization are shown in Table 3 and Figure 2. Table 3 shows all correlations with FDR<0.05; Tables S2–S6 in the Supplemental Material show complete results for the 15 most significant associations for each IQ index. The most prevalent regions were pre-central, parietal, and occipital.

**Table 3:** **Associations between functional connectivity and intelligence indices (Full-Scale IQ—FSIQ, Perceptual Organization Index—POI) for specific nodes (center coordinates in x, y, and z) in the overall network, and with (uncorrected) 95% confidence intervals**.

**Index**	**Label**	**Functional connectivity between (AAL label)**				**Correlation**	**FDR**
		**MNI Region A**	**Center A (mm)**	**MNI Region B**	**Center B (mm)**		
FSIQ	1	Fusiform R	(33.7, −40.2, −21.5)	Parietal Sup L	(−23.7, −60.8, 57.7)	0.62 (0.36, 0.80)	0.003
	2	Pre-central L	(−39.0, −7.0, 49.6)	Occipital Sup R	(24.0, −82.2, 29.3)	0.60 (0.30, 0.79)	0.05
	3	Occipital Sup R	(24.0, −82.2, 29.3)	Parietal Sup L	(−23.7, −60.8, 57.7)	0.59 (0.29, 0.79)	0.03
	4	Pre-central L	(−39.0, −7.0, 49.6)	Occipital Inf R	(37.9, −83.2, −90)	0.57 (0.26, 0.78)	0.05
POI	1	Pre-central L	(−39.0, −7.0, 49.6)	Occipital Inf L	(−36.5, −79.6, −9.2)	0.67 (0.40, 0.83)	0.006
	2	Parietal Sup R	(25.8, −60.4, 60.7)	Paracentral Lobule L	(−8.0, −26.7, 68.7)	0.66 (0.38, 0.82)	0.009
	3	Occipital Inf R	(37.9, −83.2, −90)	Post-central L	(−42.9, −23.8, 47.5)	0.63 (0.35, 0.81)	0.015
	4	Pre-central L	(−39.0, −7.0, 49.6)	Occipital Inf R	(37.9, −83.2,−90)	0.62 (0.32, 0.80)	0.015
	5	Frontal Sup Orb L	(−5.4, 52.5, −8.9)	Frontal Sup Orb R	(7.8, 50.4, −8.5)	0.61 (0.31, 0.80)	0.04
	6	Pre-central L	(−39.0, −7.0, 49.6)	Parietal Sup R	(25.8, −60.4, 60.7)	0.59 (0.29, 0.79)	0.020

*Functional connectivity was measured as the Fisher transformed correlation between the two regions' time series. Only region pairs whose connectivity was correlated with IQ index at FDR < 0.05 are shown. Tables S2–S6 in the Supplemental Material show further results*.

### Small-worldness analysis

To establish the baseline validity of the network analysis, we computed small-worldness for our data and compared the results to an independent data set. Brain networks showed a clear small-world organization over a range of thresholds. Figure 3 (left) and Figure 4 (left) show normalized characteristic path length from binary networks as a function of threshold for participants for Dataset 1 and Dataset 2, respectively. Mean values for 20 matched random networks are also shown for comparison. Figure 3 (right) and Figure 4 (right) shows the same for the normalized clustering coefficient. In both datasets, networks showed a clear small-world organization for correlation thresholds between 0.05 and 0.20, characterized by L ≈ L_random_ for thresholds lower than 0.20 and C ≫ C_random_ for thresholds higher than 0.05 (2-sample *t*-test, all *p* < α = 0.01, Bonferroni corrected for multiple thresholds).

### Associations between global network properties and intelligence

We observed a negative, though statistically weak (*p* = 0.14), correlation between FSIQ and normalized characteristic path length (lambda) (Figure 5, left). This was computed using correlation matrices binarized at a threshold of 0.45, the same threshold applied by Van den Heuvel et al. (2009), for the purpose of direct comparison.

Verbal comprehension was associated with normalized global efficiency (*r* = 0.43, *p* = 0.02, uncorrected *p*-value). Also, global efficiency was weakly correlated with FSIQ (*r* = 0.24, *p* = 0.22, uncorrected *p*-value). These results along with a complete list of correlations between intelligence scores and global network parameters are shown in Table 4.

**Table 4:** **Pearson correlations between normalized weighted-network global parameters (characteristic path length, global efficiency, and global clustering coefficient) and intelligence indices (Full-Scale IQ—FSIQ, Verbal Comprehension Index—VCI, Perceptual Organization Index—POI, Working Memory Index—WMI, Processing Speed Index—PSI) with 95% confidence intervals and *p*-values (uncorrected for multiple comparisons)**.

	**Normalized characteristic path length**	**Normalized global efficiency**	**Normalized global clustering coefficient**
FSIQ	−0.15 (−0.49, 0.22) *p* = 0.42	0.24 (−0.14, 0.56) *p* = 0.22	−0.07 (−0.42, 0.31) *p* = 0.74
VCI	−0.27 (−0.58, 0.11) *p* = 0.16	0.43 (0.08, 0.69) *p* = 0.02	−0.25 (−0.57, 0.12) *p* = 0.18
POI	−0.09 (−0.44, 0.29) *p* = 0.64	0.08 (−0.29, 0.44) *p* = 0.67	0.02 (−0.34, 0.39) *p* = 0.90
WMI	−0.08 (−0.43, 0.30) *p* = 0.68	0.17 (−0.20, 0.51) *p* = 0.36	−0.03 (−0.39, 0.34) *p* = 0.88
PSI	−0.04 (−0.40, 0.33) *p* = 0.82	0.12 (−0.26, 0.46) *p* = 0.55	0.15 (−0.23, 0.49) *p* = 0.43

### Assocations between local network properties and intelligence

We observed also possible relationships between local efficiency and measures of intelligence (Table 5, Figure 6). Prominent regions were pre-central gyrus, associated with FSIQ; caudate nucleus, associated with verbal comprehension and processing speed; bilateral inferior occipital gyrus, associated with verbal comprehension; and bilateral rolandic operculum, associated with working memory and processing speed. However, in all cases the false discovery rate was >0.05; uncorrected *p*-values are reported here.

**Table 5:** **Associations between non-normalized weighted-network local efficiency and intelligence indices (Full-Scale IQ—FSIQ, Verbal Comprehension Index—VCI, Working Memory Index—WMI, Processing Speed Index—PSI) for specific nodes in the overall network, with 95% confidence intervals and *p*-values (uncorrected for multiple comparisons)**.

**Intelligence index**	**Label**	**AAL atlas region**	**Correlation with local efficiency**
FSIQ	1	Pre-central R	0.48 (0.14, 0.72) *p* = 0.009
	2	Occipital Inf L	0.45 (0.11, 0.70) *p* = 0.013
	3	Pre-central L	0.37 (0.010, 0.65) *p* = 0.05
VCI	1	Putamen L	0.50 (0.16, 0.73) *p* = 0.006
	2	Caudate R	0.48 (0.13, 0.72) *p* = 0.009
	3	Supp Motor Area L	0.42 (0.07, 0.68) *p* = 0.022
	4	Pre-central R	0.42 (0.06, 0.68) *p* = 0.024
	5	Cingulum Mid L	0.42 (0.06, 0.68) *p* = 0.024
	6	Frontal Sup L	0.41 (0.06, 0.68) *p* = 0.026
	7	Occipital Inf R	0.41 (0.05, 0.67) *p* = 0.028
	8	Occipital Inf L	0.40 (0.04, 0.67) *p* = 0.03
	9	Caudate L	0.38 (0.016, 0.66) *p* = 0.04
	10	Parietal Sup R	0.37 (0.010, 0.65) *p* = 0.05
WMI	1	Rolandic Oper R	0.52 (0.19, 0.74) *p* = 0.004
	2	Rolandic Oper L	0.42 (0.07, 0.68) *p* = 0.022
PSI	1	Caudate L	0.46 (0.11, 0.70) *p* = 0.013
	2	Rolandic Oper L	0.45 (0.10, 0.70) *p* = 0.014
	3	Parietal Inf R	0.41 (0.05, 0.67) *p* = 0.028
	4	Caudate R	0.41 (0.05, 0.67) *p* = 0.029
	5	Temporal Mid L	0.39 (0.03, 0.66) *p* = 0.03
	6	Rolandic Oper R	0.39 (0.03, 0.66) *p* = 0.04
	7	Frontal Sup Medial L	0.39 (0.03, 0.66) *p* = 0.04
	8	Frontal Inf Tri R	0.38 (0.013, 0.65) *p* = 0.04

*Only the subset with correlations at p < 0.05 are shown (uncorrected for multiple comparisons)*.

## Discussion

We have extended a number of previous observations concerning brain functional connectivity and intelligence to the Portuguese-speaking population. These include the presence of small-world organization and correlations of intelligence with global and local characteristics of the brain's functional networks. Additionally, some novel findings in this exploratory study suggest hypotheses for future research.

The global functional brain network exhibited small-world organization at correlation thresholds between 0.05 and 0.20, α = 0.01, Bonferroni corrected for multiple comparisons of thresholds, and this closely matched the small-world organization that was apparent in the confirmation data set (Figures 3, 4). This suggests a high level of local clustering combined with a relatively small number of long-distance connections (Watts and Strogatz, 1998). This threshold range is smaller than the thresholds of 0.3–0.5 reported in previous observations of small-worldness in whole-brain networks (Van den Heuvel et al., 2008, 2009). However, node definitions differed substantially between the studies as well. Small-world networks are an attractive model for the connected human brain, because of their ability to transfer information with high efficiency for low wiring cost (Watts and Strogatz, 1998), and seem ubiquitous in the organization of anatomical connectivity, affected in a variety of diseases (Bassett and Bullmore, 2009). Moreover, Sporns and Zwi, in 2004, stated that information integration and even mental awareness depend on the small-world structure. Our replication of this effect supports the validity and the reliability of the network measures in this sample.

Globally, FSIQ showed a weak negative correlation with characteristic path length (Figure 5, left; *r* = −0.28, 95% CI = −0.59, 0.10), although with no statistical significance. Additionally, global efficiency (inversely correlated with path length) showed a weak positive correlation with FSIQ (Table 4; *r* = 0.24 95% CI = −0.14, 0.56), not statistically significant also. These same correlations were weaker when the full (weighted) association matrix was used (Table 4) instead of a binarized matrix (Figure 5). It is not known whether the thresholding step increases or decreases the reliability of the resulting measurements; however, possibly of note, correlations were observed to be the same sign in our results and in previous literature regardless of method or statistical significance. The consistent finding of a negative correlation between characteristic path length and FSIQ could be an extension to Portuguese speakers of the previous finding in Dutch speakers (Van den Heuvel et al., 2009): for characteristic path length, *r* = −0.54, 95% CI −0.80,−0.11. The negative correlation is consistent with the previously proposed idea that human intelligence is related to how efficiently different brain regions are organized and integrated (Van den Heuvel et al., 2009). It also suggests that functional brain networks are optimized in computational efficiency to promote higher processing speed (Van den Heuvel et al., 2009) with minimal wiring cost (Chklovskii et al., 2002).

The network parameters studied here were measurements of functional segregation (clustering coefficient and local efficiency), that describe the processing occurring within densely interconnected networks of brain regions; and functional integration (characteristic path length, and its inverse, global efficiency), that is related to how information from distributed brain regions is combined (Rubinov and Sporns, 2010). Global efficiency was associated with verbal comprehension (*r* = 0.43; 95% CI = 0.08, 0.69) (Table 4), a novel suggestive finding worthy of further study. This finding, combined with associations between VCI and local efficiency found in several brain regions (Table 5, Figure 6B and further discussed below) suggests that linguistic and verbal abilities are linked with a higher brain efficiency, at both global and local levels.

No other associations were found between global network parameters and intellectual performance (Table 4). Because of the relatively small sample size of this study, we are not able to make strong conclusions from this and it does not necessarily conflict with prior findings, as our estimated 95% confidence intervals included the statistically significant correlation values found by others (Song et al., 2009; Van den Heuvel et al., 2009). However, it is possible that relationships between functional connectivity and intelligence could be limited to sub-networks of the brain, rather than being present at a global level, so we proceeded to examine network characteristics at a regional level also.

Local efficiency in the caudate nuclei was associated with VCI (Table 5). Some studies show that this region is important for language and verbal abilities, revealing that a smaller shortest path between the caudate and neighbor regions would be related to a higher verbal intelligence. This was not the only feature involving the caudate that was related with verbal abilities. Caudate function has also been related to verbal fluency during a working memory task (Gruber and von Cramon, 2003), and has shown activity during speech contrasted with a non-speech rest baseline condition (Simmonds et al., 2011). Significant associations with verbal fluency performance have also been found for caudate nuclei volume, suggesting that this region is implicated in the circuitry mediating this ability (Hannan et al., 2010). Left caudate plays an important role in language selection in both monolingual and multilingual people (Crinion et al., 2006), and some studies propose that the caudate would act to fine-tune interactions between automatic and more complex language processing (Friederici, 2006) or in the resolution of word ambiguity (Ketteler et al., 2008).

Local efficiency in the parietal gyrus was correlated with Verbal Comprehension and Processing Speed indices (Table 5), and connection strengths to the parietal lobe correlated with Perceptual Organization and Working Memory indices (Tables S4, S5 in Supplemental Material).

Local efficiency and connection strength in occipital lobe regions were associated with higher general intelligence scores and other indices (Tables 3, 5). This suggests an impact of early perceptual processing on WAIS scores, especially Perceptual Organization. Although we did not observe correlations between the POI and segregational network properties (Table 5), there were some correlations with individual connections (Table 3). This may mean that this index is more related to individual connections than to network organization, possibly because of the necessity of rapid transfer of information of this region to others. It may reflect the same phenomenon observed in a recent study where higher IQ was correlated with shorter inspection time measured by EEG (which tells how fast the system extracts information from a given stimulus) because recurrent signals—those that are transmitted from a higher-tier sensory region to a lower one and that cognitive functions rely on—reach visual areas faster (Jolij et al., 2007).

Local efficiency of bilateral rolandic operculum correlated with WMI (Table 5, Figure 6C). This region encompasses part of the pre-central gyrus. This is consistent with a number of other findings relating pre-central areas to working memory, in terms of both activity (Gruber and von Cramon, 2003; Colom et al., 2010) and functional connectivity (Newton et al., 2011; Cole et al., 2012). We also observed a correlation between left pre-central regions and occipital ones with measures of general and fluid intelligence (Table 3, Figure 2A). Although other findings reported that pre-central activity and connectivity properties are related to fluid intelligence (Cole et al., 2012) as well as general intelligence (Gray et al., 2003), the specific role of the pre-central-occipital connection to the general intelligence is not known. Since these relationships are not described yet in the literature, this study may be a starting point for this question.

At the level of single paths, the strongest correlations we observed between FSIQ and functional connectivity (Table 3, Figure 2A) are consistent with the parieto-frontal integration theory (P-FIT) of Jung and Haier (2007), which was based on an extensive review of the literature relating measures of intelligence to brain structure and function. Individual differences of the described connections in this model are predicted to correlate with differences in intellectual performance. That is what we have partially observed in the patterns of functional connectivity, with higher functional connectivity predicting greater FSIQ and perceptual organization capacity. The model proposes information flow from basic sensory/perceptual processing regions to areas where structural abstraction and elaboration are involved. This is represented in our results by the connection between fusiform gyrus—a region involved in recognition of visual input and visual imagery—and parietal gyrus; and the connection between occipital and parietal cortex (Table 3). Then, a parieto-frontal network is responsible for information processing and abstraction, and finally the anterior cingulate selects the response (Jung and Haier, 2007), although no associations could be detected in our study to corroborate these two parts of the model. Nevertheless, direct connections between occipital regions and pre-central ones were associated with FSIQ (Table 3, Table S2 in Supplementary Material), which is not in accordance with the P-FIT and thus suggests a need for further study. Of note, as not all of the relationships predicted by this model were present, more experiments would be needed to robustly confirm or reject all aspects of the model.

Our selection of 82 pre-defined atlas regions as network nodes offers reduced complexity of the networks and higher data processing speed compared to a voxel-wise approach, and possibly easier interpretability of the findings in terms of known properties of the relatively large regions. The finding of small-world organization bolsters the comparability of our results to those of other studies that used different node definitions. However, it is also true that results of this study are partially dependent on the node definitions, and the node definitions used here may not coincide with others. Example of correspondences include an association between local efficiency in the left pre-central gyrus and the Full-Scale IQ for a weighted anatomical network made of 90 AAL atlas regions (Li et al., 2009) (*r* = 0.25; 0.03, 0.45), endorsing our result in Table 5 (*r* = 0.37; 0.010, 0.65). In addition, we observed a weak correlation (*r* = 0.24; *p* = 0.22) between global efficiency and Full-Scale IQ (Table 4), just as Song et al. (2009) did for the default mode network (*r* = 0.24; *p* = 0.072). Findings we did not observe include those involving local efficiency of a number of cortical and subcortical regions (Li et al., 2009) and the associations between intelligence and functional connectivity reported by Song et al. (2008, 2009). Direct comparisons are reported in the Supplement Material (Tables S7, S8).

In an exploratory study such as this one, the possibility of chance findings must be clearly communicated. Failing to acknowledge multiple tests would lead to many false positive associations. On the other hand, strictly controlling type I error is likely to eliminate interesting leads in a sample of this size. Therefore, in associations between path connectivity values and intelligence scores, we compromised by controlling the false discovery rate (estimated fraction of positive findings that were false) at 5% for each path (3321 values). As the associations with global and local network parameters showed high *p*-values, FDR control was not performed in these cases to conserve a few of the most relevant associations. Our findings that certain regions were important in more than one context, and that some regions showed symmetric bilateral effects, do lend some apparent validity to the results. We have provided complete information about the statistical reliability of all findings to facilitate hypothesis development and comparisons with other studies.

Further study of the relationships between brain network organization and intelligence would be necessary to complement and extend the findings shown here. This study considered a Portuguese-speaking population, but further data from different populations should be analyzed to allow the results to be generalized, in particular the relationship between global efficiency and verbal intelligence that was strongly apparent in our work. More detailed templates could be used in the definition of the network nodes for a finer-grained investigation of the brain's connectivity. It is also noteworthy that we considered only positive correlations between nodes; anticorrelations may provide complementary data once methods to quantify them arise (Rubinov and Sporns, 2010).

The findings shown here replicate and extend the negative association between characteristic path length of the functional brain network and cognitive general intelligence for a Portuguese-speaking population. The small-world organization model was verified as a feature of brain networks, suggesting an ability to transfer information with high efficiency and low wiring cost. Global efficiency was weakly associated with general intelligence but strongly associated with VCI, a novel finding. Combined with the observed relationship between verbal comprehension and local efficiency in several regions, this suggests that a possible link between language ability and organizational and integrational properties of the brain network warrants further study. Additionally, an exploratory analysis suggested associations between intelligence and network properties of frontal, parietal, and occipital cortices; and fusiform, supramarginal, pre-central gyrus, and caudate nuclei.

### Conflict of interest statement

The authors declare that the research was conducted in the absence of any commercial or financial relationships that could be construed as a potential conflict of interest.
