# Supplementary material for: Analyzing the association between functional connectivity of the brain and intellectual performance
Source: Front Hum Neurosci. 2015 Feb 10;9:61. doi: 10.3389/fnhum.2015.00061 (PMC4322636; doi:10.3389/fnhum.2015.00061)
Supplement: Supplementary file 1 [file DataSheet1.PDF]

Supplementary Material

Analyzing the association between functional connectivity of the brain and intellectual performance

Pamplona, G. S. P.[1]; Santos Neto, G. S.[2]; Rosset, S. R. E.[2] ; Rogers, B. P.[3]; Salmon, C. E. G.[1]

[1]InBrain Lab, Department of Physics, Faculty of Philosophy, Sciences and Letters of Ribeirão Preto, University of São Paulo, Ribeirão Preto, SP, Brazil.
[2]Faculty of Medicine of Ribeirão Preto, University of São Paulo, Ribeirão Preto, SP, Brazil.
[3]Institute of Imaging Science, Vanderbilt University, Nashville, TN, USA.

***Corresponding author at:** University of São Paulo, Faculty of Philosophy, Sciences and Letters of Ribeirão Preto, Department of Physics, InBrain Lab, Av. Bandeirantes 3900, Ribeirão Preto, SP, 14040-900, Brazil.
gustavopamplona@usp.br

Brain regions	AAL label	X (mm)	Y (mm)	Z (mm)
Precentral gyrus	Precentral L	-39,0	-7,0	49,6
	Precentral R	41,2	-9,6	50,8
Superior frontal gyrus, dorsolateral	Frontal Sup L	-18,8	33,4	41,0
	Frontal Sup R	21,6	29,9	42,5
Superior frontal gyrus, orbital part	Frontal Sup Orb L	-16.8	45.9	-14.7
	Frontal Sup Orb R	18.2	46.6	-15.5
Middle frontal gyrus	Frontal Mid L	-33.8	31.5	34.1
	Frontal Mid R	37.4	31.8	32.8
Middle frontal gyrus, orbital part	Frontal Mid Orb L	-30.9	49.1	-11.0
	Frontal Mid Orb R	32.9	51.3	-12.1
Inferior frontal gyrus, opercular part	Frontal Inf Oper L	-48.8	11.5	17.8
	Frontal Inf Oper R	49.9	13.7	20.2
Inferior frontal gyrus, triangular part	Frontal Inf Tri L	-45.9	28.7	12.6
	Frontal Inf Tri R	50.1	28.9	12.8
Inferior frontal gyrus, orbital part	Frontal Inf Orb L	-36.2	29.5	-13.5
	Frontal Inf Orb R	40.9	31.0	-13.3
Rolandic operculum	Rolandic Oper L	-47.4	-9.8	12.6
	Rolandic Oper R	52.4	-7.5	13.3
Supplementary motor area	Supp Motor Area L	-5.7	3.6	60.1
	Supp Motor Area R	8.2	-1.1	60.5
Superior frontal gyrus, medial	Frontal Sup Medial L	-5.2	47.9	29.6
	Frontal Sup Medial R	8.8	49.5	28.9
Superior frontal gyrus, medial orbital	Frontal Med Orb L	-5.4	52.5	-8.9
	Frontal Med Orb R	7.8	50.4	-8.5
Gyrus rectus	Rectus L	-5.4	35.7	-19.6
	Rectus R	8.0	34.5	-19.4
Insula	Insula L	-35.4	5.4	2.2
	Insula R	38.7	5.0	0.8
Anterior cingulate and paracingulate gyri	Cingulum Ant L	-4.4	34.2	12.5
	Cingulum Ant R	8.1	35.7	14.4

Median cingulate and paracingulate gyri	Cingulum Mid L	-5.9	-16.1	40.2
	Cingulum Mid R	7.7	-10.2	38.4
Posterior cingulate gyrus	Cingulum Post L	-5.2	-44.2	23.3
	Cingulum Post R	7.2	-43.1	20.5
Hippocampus	Hippocampus L	-25.3	-22.0	-11.4
	Hippocampus R	28.9	-21.0	-11.6
Parahippocampal gyrus	ParaHippocampal L	-21.5	-17.3	-21.9
	ParaHippocampal R	25.1	-16.3	-21.7
Calcarine fissure and surrounding cortex	Calcarine L	-7.5	-79.8	5.1
	Calcarine R	15.7	-74.4	8.0
Cuneus	Cuneus L	-6.3	-81.4	25.8
	Cuneus R	13.2	-80.6	26.9
Lingual gyrus	Lingual L	-14.9	-68.9	-6.0
	Lingual R	16.1	-68.1	-5.2
Superior occipital gyrus	Occipital Sup L	-16.8	-85.6	26.9
	Occipital Sup R	24.0	-82.2	29.3
Middle occipital gyrus	Occipital Mid L	-32.6	-82.0	14.8
	Occipital Mid R	37.1	-81.0	18.1
Inferior occipital gyrus	Occipital Inf L	-36.5	-79.6	-9.2
	Occipital Inf R	37.9	-83.2	-9.0
Fusiform gyrus	Fusiform L	-31.4	-41.4	-21.6
	Fusiform R	33.7	-40.2	-21.5
Postcentral gyrus	Postcentral L	-42.9	-23.8	47.5
	Postcentral R	41.2	-26.8	51.2
Superior parietal gyrus	Parietal Sup L	-23.7	-60.8	57.7
	Parietal Sup R	25.8	-60.4	60.7
Inferior parietal, but supramarginal and angular gyri	Parietal Inf L	-43.1	-47.0	45.4
	Parietal Inf R	46.3	-47.6	48.2
Supramarginal gyrus	SupraMarginal L	-56.1	-34.9	29.1
	SupraMarginal R	57.3	-32.8	33.1
Angular gyrus	Angular L	-44.4	-62.1	34.3
	Angular R	45.2	-61.2	37.3
Precuneus	Precuneus L	-7.6	-57.3	46.6
	Precuneus R	9.7	-57.3	42.4
Paracentral lobule	Paracentral Lobule L	-8.0	-26.7	68.7
	Paracentral Lobule R	7.1	-32.9	66.8
Caudate nucleus	Caudate L	-11.8	9.7	8.1
	Caudate R	14.5	10.8	8.1
Lenticular nucleus, putamen	Putamen L	-24.2	2.6	1.1
	Putamen R	27.5	3.7	1.2
Thalamus	Thalamus L	-11.2	-18.8	6.6
	Thalamus R	12.7	-18.8	6.7
Superior temporal gyrus	Temporal Sup L	-53.4	-22.0	5.8
	Temporal Sup R	57.8	-23.0	5.4
Temporal pole: superior temporal gyrus	Temporal Pole Sup L	-40.2	13.9	-21.4
	Temporal Pole Sup R	47.9	13.5	-18.2
Middle temporal gyrus	Temporal Mid L	-55.9	-35.0	-3.6
	Temporal Mid R	57.2	-38.6	-2.8
Temporal pole: middle temporal gyrus	Temporal Pole Mid L	-36.7	13.3	-35.4
	Temporal Pole Mid R	44.0	13.2	-33.5

Inferior temporal gyrus	Temporal Inf L	-50.0	-29.3	-24.5
	Temporal Inf R	53.4	-32.1	-23.7

Table S1. 82 cortical and subcortical regions with at least 300 voxel in AAL space defined using the automated anatomical labeling atlas (AAL), with their AAL abbreviations and the locations of their centers, in x, y, and z.

Ranking	Functional connectivity between (AAL label)		Correlation	Corrected p-value
1	Fusiform R	Parietal Sup L	0.62 (0.36,0.80) p=0.0004	0.003
2	Precentral L	Occipital Sup R	0.60 (0.30,0.79) p=0.0006	0.05
3	Occipital Sup R	Parietal Sup L	0.59 (0.29,0.79) p=0.0008	0.03
4	Precentral L	Occipital Inf R	0.57 (0.26,0.78) p=0.0012	0.05
5	Thalamus L	Temporal Pole Sup R	0.54 (0.21,0.76) p=0.0027	0.22
6	Precentral R	Lingual L	0.54 (0.21,0.75) p=0.0027	0.19
7	Precentral L	Occipital Inf L	0.53 (0.21,0.75) p=0.0028	0.06
8	Precentral L	Occipital Mid R	0.53 (0.21,0.75) p=0.0029	0.06
9	Occipital Inf R	Postcentral L	0.52 (0.19,0.75) p=0.004	0.14
10	Cingulum Mid L	SupraMarginal L	0.52 (0.19,0.75) p=0.004	0.29
11	Calcarine R	Parietal Sup L	0.52 (0.19,0.75) p=0.004	0.3
12	Lingual L	Paracentral Lobule L	0.52 (0.19,0.75) p=0.004	0.10
13	Precuneus L	Thalamus R	0.52 (0.19,0.74) p=0.004	0.19
14	Insula L	Paracentral Lobule R	0.51 (0.18,0.74) p=0.005	0.4
15	Lingual L	Parietal Sup R	0.51 (0.18,0.74) p=0.005	0.10

Table S2. Associations between strength of functional connectivity with FSIQ, with 95% confidence intervals and p-values (uncorrected and corrected for multiple comparisons). The fifteen highest correlations are shown in descending order. Regions marked with (*) are in according to (13).

Ranking	Functional connectivity between (AAL label)		Correlation	Corrected p-value
1	Temporal Mid R	Temporal Inf L	-0.60 (-0.79,-0.29) p=0.0007	0.05
2	Cingulum Post L	Caudate R	0.59 (0.28,0.78) p=0.0008	0.07
3	Rectus L	Putamen R	0.59 (0.28,0.78) p=0.0008	0.07
4	Frontal Sup L	Caudate R	0.56 (0.25,0.77) p=0.0014	0.08
5	Precentral R	Occipital Mid L	0.56 (0.24,0.77) p=0.0016	0.08
6	Frontal Sup Medial L	Caudate R	0.56 (0.24,0.77) p=0.0017	0.09
7	Frontal Sup L	Caudate L	0.55 (0.23,0.76) p=0.0020	0.08
8	Frontal Sup Medial L	Caudate L	0.55 (0.22,0.76) p=0.0022	0.09
9	Rectus R	Putamen R	0.54 (0.22,0.76) p=0.0024	0.20
10	Precentral R	Lingual L	0.53 (0.21,0.75) p=0.003	0.08
11	Precentral R	Calcarine R	0.53 (0.21,0.75) p=0.003	0.08
12	Fusiform L	Postcentral R	0.53 (0.20,0.75) p=0.003	0.26
13	Supp Motor Area R	Occipital Mid R	0.52 (0.19,0.75) p=0.004	0.22
14	Frontal Inf Orb L	Supp Motor Area L	0.51 (0.18,0.74) p=0.004	0.28
15	Postcentral L	Temporal Sup R	0.51 (0.18,0.74) p=0.005	0.4

Table S3. Associations between strength of functional connectivity with VCI, with 95% confidence intervals and p-values (uncorrected and corrected for multiple comparisons). The fifteen highest correlations are shown in descending order. Regions marked with (*) are in according to (13).

Ranking	Functional connectivity between (AAL label)		Correlation	Corrected p-value
1	Precentral L	Occipital Inf L	0.67 (0.40,0.83) p=0.00008	0.006
2	Parietal Sup R	Paracentral Lobule L	0.66 (0.38,0.82) p=0.00011	0.009
3	Occipital Inf R	Postcentral L	0.63 (0.35,0.81) p=0.00022	0.015
4	Precentral L	Occipital Inf R	0.62 (0.32,0.80) p=0.0004	0.015
5	Frontal Sup Orb L	Frontal Sup Orb R	0.61 (0.31,0.80) p=0.0005	0.04
6	Precentral L	Parietal Sup R	0.59 (0.29,0.79) p=0.0007	0.020
7	Rectus R	Precuneus R	-0.57 (-0.78,-0.26) p=0.0011	0.09
8	Frontal Sup Orb L	Rectus L	0.57 (0.25,0.77) p=0.0013	0.05
9	Occipital Inf R	Postcentral R*	0.55 (0.23,0.76) p=0.0019	0.05
10	Lingual L	Putamen R	0.55 (0.23,0.76) p=0.0021	0.13
11	Precentral R	Frontal Inf Oper L	0.53 (0.20,0.75) p=0.003	0.25
12	Temporal Sup R*	Temporal Mid R*	-0.52 (-0.75,-0.20) p=0.004	0.28
13	Rectus R	Precuneus L	-0.52 (-0.74,-0.19) p=0.004	0.16
14	Precentral L	Occipital Sup R	0.52 (0.19,0.74) p=0.004	0.08
15	Lingual L	Putamen L	0.52 (0.18,0.74) p=0.004	0.13

Table S4. Associations between strength of functional connectivity with POI, with 95% confidence intervals and p-values (uncorrected and corrected for multiple comparisons). The fifteen highest correlations are shown in descending order. Regions marked with (*) are in according to (13).

Ranking	Functional connectivity between (AAL label)		Correlation	Corrected p-value
1	Frontal Mid R	Frontal Mid Orb R	-0.58 (-0.78,-0.28) p=0.0009	0.07
2	Frontal Mid Orb R	Cingulum Mid R	-0.56 (-0.77,-0.25) p=0.0014	0.06
3	Frontal Sup Orb L	Parietal Sup R	-0.54 (-0.76,-0.22) p=0.0023	0.19
4	Rolandic Oper R	Temporal Mid R	-0.54 (-0.76,-0.22) p=0.0025	0.15
5	Precuneus R	Thalamus R	0.53 (0.21,0.75) p=0.0029	0.20
6	Fusiform R	Parietal Sup L	0.53 (0.20,0.75) p=0.003	0.23
7	Precentral L*	Occipital Mid R	0.53 (0.20,0.75) p=0.003	0.24
8	Frontal Sup Orb R	Parietal Sup R	-0.53 (-0.75,-0.20) p=0.003	0.18
9	Frontal Sup L	Temporal Mid R	0.52 (0.19,0.75) p=0.004	0.26
10	Frontal Sup L	Temporal Mid L*	0.52 (0.19,0.74) p=0.004	0.16
11	SupraMarginal R	Caudate L	-0.51 (-0.74,-0,18) p=0.004	0.16
12	Precentral L*	Occipital Mid L	0.51 (0.18,0,74) p=0.004	0.3
13	Cingulum Ant R	Occipital Mid R	-0.50 (-0.71,-0.17) p=0.005	0.18
14	ParaHippocampal R	Fusiform R	0.50 (0.16,0.73) p=0.006	0.4
15	Rolandic Oper L	Temporal Mid R	-0.49 (-0.73,-0.16) p=0.007	0.5

Table S5. Associations between strength of functional connectivity with WMI, with 95% confidence intervals and p-values (uncorrected and corrected for multiple comparisons). The fifteen highest correlations are shown in descending order. Regions marked with (*) are in according to (13).

Ranking	Functional connectivity between (AAL label)		Correlation	Corrected p-value
1	Frontal Mid R	Frontal Mid Orb R	-0.58 (-0.78,-0.28) p=0.0009	0.07
2	Frontal Mid Orb R	Cingulum Mid R	-0.56 (-0.77,-0.25) p=0.0014	0.06
3	Frontal Sup Orb L	Parietal Sup R	-0.54 (-0.76,-0.22) p=0.0023	0.19
4	Rolandic Oper R	Temporal Mid R	-0.54 (-0.76,-0.22) p=0.0025	0.15
5	Precuneus R	Thalamus R	0.53 (0.21,0.75) p=0.0029	0.20
6	Fusiform R	Parietal Sup L	0.53 (0.20,0.75) p=0.003	0.23

7	Precentral L*	Occipital Mid R	0.53 (0.20,0.75) p=0.003	0.24
8	Frontal Sup Orb R	Parietal Sup R	-0.53 (-0.75,-0.20) p=0.003	0.18
9	Frontal Sup L	Temporal Mid R	0.52 (0.19,0.75) p=0.004	0.26
10	Frontal Sup L	Temporal Mid L*	0.52 (0.19,0.74) p=0.004	0.16
11	SupraMarginal R	Caudate L	-0.51 (-0.74,-0,18) p=0.004	0.16
12	Precentral L*	Occipital Mid L	0.51 (0.18,0,74) p=0.004	0.3
13	Cingulum Ant R	Occipital Mid R	-0.50 (-0.71,-0.17) p=0.005	0.18
14	ParaHippocampal R	Fusiform R	0.50 (0.16,0.73) p=0.006	0.4
15	Rolandic Oper L	Temporal Mid R	-0.49 (-0.73,-0.16) p=0.007	0.5

Table S6. Associations between strength of functional connectivity with PSI, with 95% confidence intervals and p-values (uncorrected and corrected for multiple comparisons). The fifteen highest correlations are shown in descending order. Regions marked with (*) are in according to (13).

Connected regions (Song et al., 2008)		Approximate AAL regions		Correlation (Song et al., 2008) (p<0.01)	Correlation found in our study
L.DLPFC	R.DLPFC	Precentral L	Precentral R	0.47	0.37 (0.009,0.65) p=0.05
	R.IFG/MFG		Frontal Mid R	0.44	0.24 (-0.14,0.56) p=0.20
	L.SFG/MPFG		Frontal Mid Orb L	-0.48	0.26 (-0.12,0.57) p=0.17
	R.SFG/MPFG		Frontal Mid Orb R	-0.36	-0.06 (-0.42,0.31) p=0.7
	R.IPL		Parietal Inf R	0.44	0.23 (-0.15,0.55) p=0.22
	B.LG		Lingual L	-0.42	0.43 (0.08,0.69) p=0.019
			Lingual R		0.42 (0.06,0.68) p=0.024
	B.Cu		Cuneus L	-0.38	0.18 (-0.20,0.51) p=0.4
			Cuneus R		0.37 (0.004,0.65) p=0.05
	R.HP		Hippocampus R	0.49	-0.08 (-0.44,0.29) p=0.7
R.DLPFC	R.IPL	Precentral R	Parietal Inf R	0.41	0.26 (-0.11,0.58) p=0.17
	B.LG		Lingual L	-0.45	0.54 (0.21,0.75) p=0.0027
			Lingual R		0.49 (0.15,0.73) p=0.007
	L.Cu		Cuneus L	-0.42	0.35 (-0.015,0.64) p=0.06

Table S7. Comparison between the results of correlations between strength of functional connections and FSIQ from Song et al. (2008) and our study. In Song et al., the size of each region varied among 20 and 112 voxels of 3x3x3 mm. In our study, the regions were defined by the AAL atlas. p-value is uncorrected. Abbreviations: L - left, R - right, B - bilateral, DLPFC - dorsolateral prefrontal cortex, MFG - middle frontal gyrus, IFG - inferior frontal gyrus, MPFG - medial prefrontal gyrus, SFG - superior frontal gyrus, IPL - inferior parietal lobules, LG - lingual gyrus, Cu - cuneus, HP - hippocampus.

Connected regions (Song et al., 2009)		Approximate AAL regions		Correlation (Song et al., 2009) (p<0.05)	Correlation found in our study
vMPFC	PCC	Frontal Med Orb L	Cingulum Post L	0.26	0.16 (-0.22,0.50) p=0.4
Rsp	L.PHC	Calcarine R	Parahippocampal L	0.36	0.08 (-0.30,0.43) p=0.7
R.IT	L.PHC	Temporal Mid R	Parahippocampal L	0.33	0.16 (-0.22,0.50) p=0.4
Rsp	vMPFC	Calcarine R	Frontal Med Orb L	0.27	-0.22 (-0.54,0.16) p=0.26
Rsp	R.PHC	Calcarine R	Parahippocampal R	0.26	0.28 (-0.10,0.59) p=0.14

Table S8. Comparison between the results of correlations between strength of functional connections and FSIQ from Song et al. (2009) and our study. In Song et al., the size of each region was 33 voxels of 3x3x3 mm. In our study, the regions were defined by the AAL atlas. p-value is uncorrected. Abbreviations: L - left, R - right, vMPFC - medial prefrontal cortex (ventral), PCC - posterior cingulate cortex, Rsp - retrosplenial, PHC - parahippocampal gyrus, IT - inferior temporal cortex.
